# Investigation of the stress response to mechanical *versus* thermal non-ablative focused ultrasound therapy in three *in vivo* murine cancer models

**DOI:** 10.1186/2050-5736-3-S1-P71

**Published:** 2015-06-30

**Authors:** Steffie Peters, Karin Skalina, Lisa Scandiuzzi, Ari Partanen, Holger Grüll, Chandan Guha

**Affiliations:** 1 Eindhoven University of Technology, Eindhoven, Netherlands; 2 Albert Einstein College of Medicine, New York, New York, United States; 3 Philips Healthcare, Bethesda, Maryland, United States; 4 Montefiore Medical Center, New York, New York, United States

## Background/introduction

Within the focal point of focused ultrasound (FUS), high pressure fluctuations cause shear stress on cells and tissues with energy dissipation leading to heating of the targeted tissue. The extent of the mechanical and thermal effects strongly depends on the ultrasound parameters and also potentially the acoustic properties of the tumor. High temperatures lead to coagulative necrosis with little immunogenic response as tumor antigens most likely denature during the ablation. As antigen presentation by activated dendritic cells (DCs) is necessary to activate T cells during an anti-tumor immune response, we hypothesize that non-ablative FUS treatment can be used to increase tumor immunogenicity by activating molecules that enhance intratumor dendritic cell (DC) infiltration and T cell activation. One of the first steps in this process is an activated stress response, including surface calreticulin (CRT), high mobility group protein B1 (HMGB1) release, ATP secretion and heat shock protein 70 (HSP70) surface expression and release. Using non-ablative FUS, antigen release can be stimulated by mechanical stress, thermal stress, or both. Here, we examine the stress response following non-ablative FUS treatment in three murine tumor models with varying consistencies and determine the causative agent (mechanical or thermal energy) of the stress.

## Methods

We tested a range of non-ablative FUS treatment protocols *in vivo* in three subcutaneous tumor models in C57BL/6 mice, varying the mechanical and thermal energies to determine which protocol results in the greatest anti-tumor immunity. We investigated CRT surface expression, ATP release and both intra and extracellular expression/release of HMGB1 and HSP70. Since the effect of FUS treatment may vary depending on the cancer cell type, we investigated three different mouse cancer cell lines varying in consistency from solid to fluid [TPSA23 (prostate), 3LL (Lewis lung), B16F10 (melanoma)]. Animals were sacrificed 24 hours post-treatment and tumors were excised for stress response analysis by flow cytometry. Plasma was also obtained for analysis of soluble proteins by ELISA. Stress response was compared to non-treated tumors (control) as well as to *in vitro* treatment of a cell pellet with the same FUS parameters. For all FUS treatment a Philips Therapy and Imaging Probe System (TIPS, Philips Research Briarcliff, USA) was used.

## Results and conclusions

**Figure 1:**
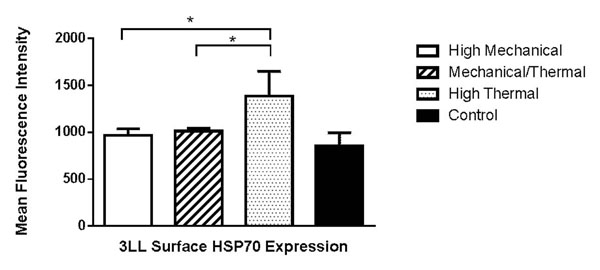
Mean Fluorescence Intensity for surface HSP70 in 3LL for different FUS settings. High thermal settings show significant increase compared to control as well as other FUS settings

While experiments are still ongoing, we have observed a significant increase (One-way ANOVA p < 0.01) in surface expression of HSP70 in an *in vivo* model of 3LL 24 hours following FUS treatment that delivers high thermal energy when compared to other treatment settings and no treatment (Figure 1). Future experiments will determine whether there are increased soluble HSP70, HMGB1, and ATP levels with mechanical or thermal stress.

